# Current immunotherapeutic approaches to diffuse intrinsic pontine glioma

**DOI:** 10.3389/fgene.2024.1349612

**Published:** 2024-05-07

**Authors:** Catherine Lin, Christian Smith, James Rutka

**Affiliations:** ^1^ Cell Biology Research Program, The Hospital for Sick Children, Toronto, ON, Canada; ^2^ Arthur and Sonia Labatt Brain Tumour Research Centre, The Hospital for Sick Children, Toronto, ON, Canada; ^3^ Department of Laboratory Medicine and Pathobiology, University of Toronto, Toronto, ON, Canada; ^4^ Division of Neurosurgery, The Hospital for Sick Children, Toronto, ON, Canada; ^5^ Division of Neurosurgery, Department of Surgery, University of Toronto, Toronto, ON, Canada

**Keywords:** glioma, diffuse intrinsic pontine glioma (DIPG), immunotherapy, brainstem, blood-brain barrier (BBB)

## Abstract

Diffuse intrinsic pontine glioma (DIPG) is an aggressive brain tumour that occurs in the pons of the brainstem and accounts for over 80% of all brainstem gliomas. The median age at diagnosis is 6–7 years old, with less than 10% overall survival 2 years after diagnosis and less than 1% after 5 years. DIPGs are surgically inaccessible, and radiation therapy provides only transient benefit, with death ensuing from relentless local tumour infiltration. DIPGs are now the leading cause of brain tumour deaths in children, with a societal cancer burden in years of life lost (YLL) of more than 67 per individual, *versus* approximately 14 and 16 YLL for lung and breast cancer respectively. More than 95 clinical drug trials have been conducted on children with DIPGs, and all have failed to improve survival. No single or combination chemotherapeutic strategy has been successful to date because of our inability to identify targeted drugs for this disease and to deliver these drugs across an intact blood-brain barrier (BBB). Accordingly, there has been an increased focus on immunotherapy research in DIPG, with explorations into treatments such as chimeric antigen receptor T (CAR-T) cells, immune checkpoint blockades, cancer vaccines, and autologous cell transfer therapy. Here, we review the most recent advances in identifying genetic factors influencing the development of immunotherapy for DIPG. Additionally, we explore emerging technologies such as Magnetic Resonance-guided Focused Ultrasound (MRgFUS) in potential combinatorial approaches to treat DIPG.

## Introduction

Diffuse intrinsic pontine glioma (DIPG) is a brain tumour occurring in the pons of the brainstem and accounts for over 80% of all brainstem gliomas (Srikanthan et al., 2021). DIPGs are a subset of diffuse midline gliomas (DMG) and are characterized by the lysine 27 to methionine (K27M) mutation on histone 3 (H3K27M) (International Agency for Research on Cancer, 2022). The median age at diagnosis is 6–7 years old, with less than 1% survival after 5 years (Johung and Monje, 2017; Srikanthan et al., 2021). Its location in the pons makes it impossible to resect the tumour, with chemotherapy and radiotherapy providing only transient benefits. More than 95 clinical drug trials have been conducted on children with DIPGs, and all have failed to improve survival. No single or combination chemotherapeutic strategy has been successful to date because of our inability to identify targeted drugs for this disease and to deliver these drugs across an intact blood-brain barrier (BBB).

**FIGURE 1:**
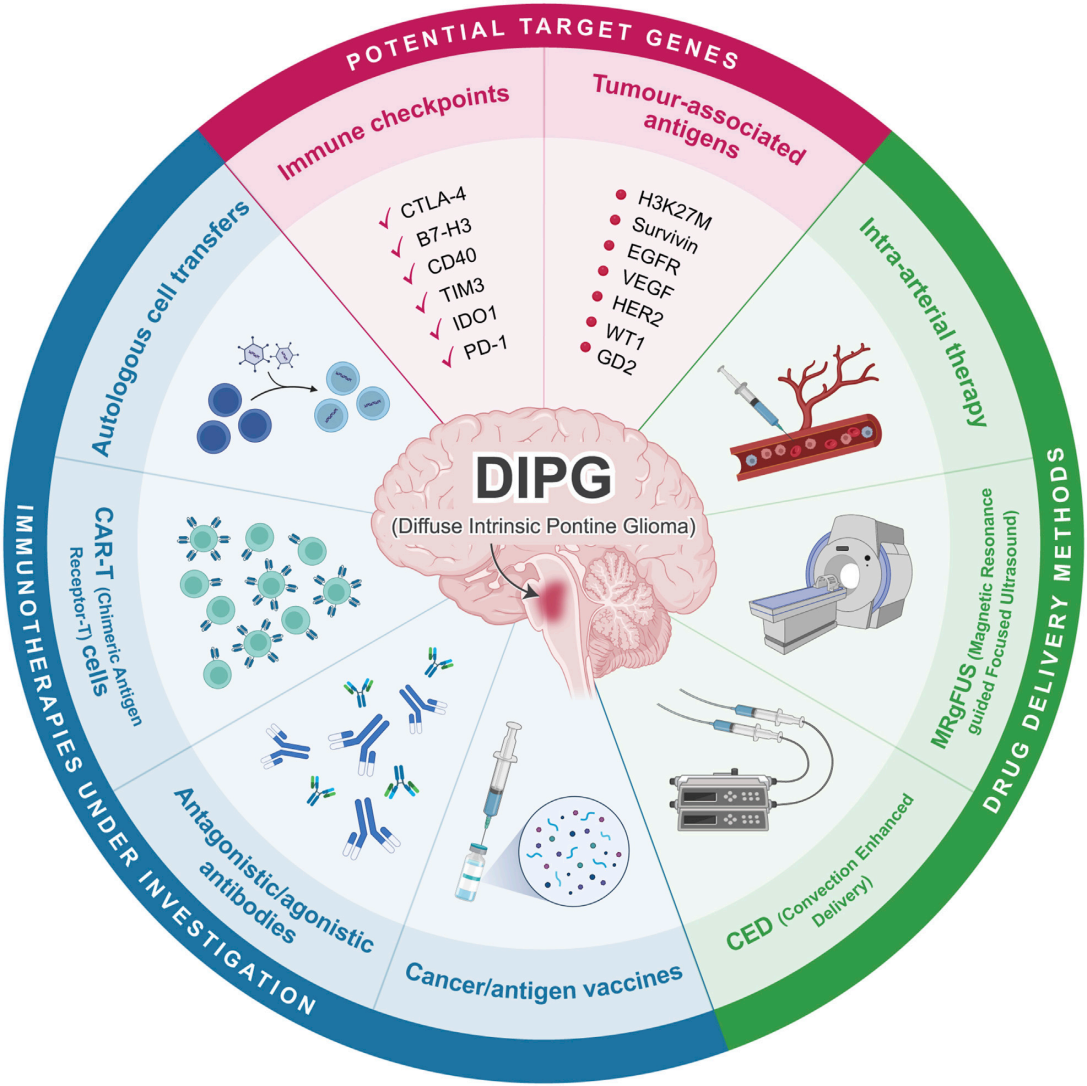
Overview of current immunotherapeutic approaches in DIPG. Cancer vaccines, antibodies, CAR-T cells, and autologous cell transfers are currently being investigated as possible immunotherapy for DIPG. Potential target genes include various immune checkpoints and tumour associated antigens. Drug delivery can be conducted through CED, MRgFUS, or intra-arterial therapy. Created with BioRender.com.

Recent advances in immunology have allowed for improvements to cancer treatment. Immunotherapy focuses on harnessing the individual’s immune system to eradicate the tumour, with the characteristics of the tumour-immune microenvironment (TIME) playing a major role in the efficacy of these therapeutics (Waldman et al., 2020). Tumours with higher numbers of immune cells are called “immune-hot” and generally respond better to immunotherapy. In contrast, those with a low immune cell population are “immune-cold,” and immunotherapy has minimal effects as these treatments rely on immune activation to clear the tumours (Galon and Bruni, 2019). Past studies have shown DIPG as an “immune-cold” tumour, characterized by a low population of immune cells and reduced expression of immune checkpoint molecules, which poses a significant challenge in immunotherapy treatments in DIPG (Figure 1). In a healthy individual, the balance between inhibitory and stimulatory immune checkpoint pathways is maintained such that inhibitory pathways support self-tolerance and immunosuppression, while stimulatory pathways are focused on activating the immune system against foreign antigens (Marin-Acevedo et al., 2018; Zhang and Zheng, 2020). In a tumour context, the downregulation of immune stimulatory signals and upregulation of suppressive signals mediated by these checkpoints reduces anti-tumour activity (He and Xu, 2020; Zhang and Zheng, 2020). By modulating the signalling of immune checkpoints, overall immune activation can be increased to kill cancer cells.

### Immune checkpoint approaches

In DIPG patients, Hwang et al. (2018) found that inhibition of PD-1, an immunosuppressive checkpoint molecule, using pembrolizumab resulted in a worsened condition, while Cacciotti et al. (2020) and Dunkel et al. (2023) (NCT03130959) found no significant improvements in survival as well as minimal to no adverse effects. Overall results for the use of anti-PD1 in DIPG do not seem promising (Hwang et al., 2018; Cacciotti et al., 2020; Dunkel et al., 2023). Recent studies have also investigated other checkpoint molecules such as TIM3, B7-H3, and CD40. TIM3 is an inhibitory checkpoint expressed on DIPG cells and immune cells such as microglia and macrophages. Ausejo-Mauleon et al. (2023) reported that using TIM-3 blockades promotes anti-tumour effects in immunocompetent DIPG murine models and found enhanced microglia- and CD8^+^ T cell-mediated pro-inflammatory immune responses, which ultimately improved survival (Ausejo-Mauleon et al., 2023). The differences in efficacy between anti-PD1 and anti-TIM3 may be attributed to the tumour-immune microenvironment (TIME) composition. Past research indicates that the DIPG TIME has low PD-1 expression, while there is a comparatively higher TIM3 expression (Lin et al., 2018; Ausejo-Mauleon et al., 2023; Chen et al., 2023). This highlights the role that macrophages may play in the TIME and is a potential area for exploration (Lin et al., 2018; Lieberman et al., 2019). B7-H3, also known as CD276, is expressed on both tumour and antigen-presenting cells and inhibits the anti-tumour functions of T cells (Zhang and Zheng, 2020). In the DIPG context, studies have utilized convection-enhanced delivery in combination with the monoclonal B7-H3 antibody, 8H9, as well as the development of anti-B7-H3 CAR-T cells (Souweidane et al., 2018; Vitanza et al., 2023) (NCT04185038). Clinical trials are currently ongoing for various forms of B7-H3-targeting CAR-T cells, such as the single target B7-H3 and the quad-target B7-H3, EGFR806, HER2, and IL13-Zetakine (Quad) CAR-T Cell (NCT04185038, NCT05768880). Preliminary results of the anti-B7-H3 CAR-T cell study showed improved survival (Vitanza et al., 2023). Extended survival was also observed in the 8H9 antibody study, although selection bias may be present due to the study’s design (Souweidane et al., 2018).

The indoleamine 2,3-dioxygenase (IDO) pathway is primarily involved in the conversion of tryptophan into kynurenines, but indoleamine 2,3-dioxygenase 1 (IDO1), an enzyme in the pathway, also acts as an immune checkpoint by modulating immunosuppressive responses (Zhai et al., 2018). A clinical trial (NCT02502708) evaluating indoximod, an IDO pathway inhibitor, combined with the chemotherapy drug temozolomide and radiation recently published its results. Overall, the authors concluded that the combination treatment can be tolerated, with variable disease and immune responses observed among the participants. Further studies must be conducted to evaluate treatment efficacy (Johnson et al., 2023). Contrary to the previously mentioned immune checkpoints, CD40 is typically involved in the stimulatory pathway and is expressed on immune cells to promote tumour-killing activity. An agonistic CD40 antibody, APX005M, has been developed to enhance immune activity against the tumour and induce tumour cell apoptosis. Studies have confirmed preliminary safety, with efficacy against DIPG being tested (Lindsay et al., 2020) (NCT03389802).

### Targeting of tumour-associated antigens

Tumour-associated antigens (TAAs) are also potential targets in immunotherapy, ranging from antigens seen across different cancer types to DIPG-specific TAAs. GD2 is a disialoganglioside that is highly expressed in solid tumours, including DIPG, small cell lung cancer, and melanoma; GD2 expression in normal tissue is limited, making GD2 an ideal target for immunotherapy with minimal off-target effects (Mount et al., 2018; Nazha et al., 2020). Clinical trials are ongoing for anti-GD2 CAR-T cells (NCT04196413) and C7R-coexpressing anti-GD2 CAR-T cells (NCT04099797), with clinical and radiological improvements observed in DIPG/DMG patients enrolled in the anti-GD2 CAR-T cells trial (Majzner et al., 2022). Anti-GD2 CAR-T cells and anti-GD2 CAR NK-92 cells are effective in *in vivo* assays using patient-derived DIPG cells and DIPG cell lines (Mount et al., 2018; Mount et al., 2018; Zuo et al., 2023). The Wilms’ tumor 1 gene (WT1) is an oncogene in various cancers including leukemia, breast cancer, glioblastoma, and DIPG. Overexpression of WT1 is associated with these cancers, and targeted protein knockdown inhibits cancer growth (Qi et al., 2015). A completed clinical trial that studied DSP-7888, a cancer vaccine that induces WT1-specific T cells, showed improved survival in DIPG patients compared to controls (Fujisaki et al., 2018) (NCT02750891). Separately, an ongoing clinical trial investigates vaccination with WT1 mRNA-loaded autologous monocyte-derived dendritic cells (DCs) as a potential therapeutic for DIPG. It is hypothesized that the DC vaccine will act as an adjuvant to boost anti-tumour immune activity with minimal side effects, given its specificity to WT1-expressing cells (NCT04911621). Epidermal growth factor (EGFR) is another common TAA expressed in tumour cells with limited expression in normal tissue. EGFR mutations leads to constitutive activation of the gene and results in cancer phenotypes, with EGFR overexpression observed in DIPG (Li et al., 2012). More recent studies focus on using nimotuzumab, an anti-EGFR, combined with radiation or radiochemotherapy (Fleischhack et al., 2019) (NCT04532229). While radiation with nimotuzumab does not significantly improve survival compared to radiochemotherapy alone, side effects and adverse events were significantly reduced. Overall, the DIPG patients had an improved quality of life, albeit no changes in survival (Fleischhack et al., 2019). The HER2 protein is a member of the EGFR family, and its overexpression is seen in many solid tumours, including DIPG, with breast cancer being the most notable (Oh and Bang, 2020). Wang et al. (2023a) investigated the use of anti-HER2 CAR-T cells as a therapeutic in DIPG and other DMGs. Results showed HER2-specific immune targeting and cytokine release when co-cultured *in vitro* with patient-derived DIPG cells. Anti-HER2 CAR-T cells also reduce tumour size in *in vivo* DIPG xenografts. The authors concluded that the CAR-T cells may show efficacy in DIPG patients, although a clinical trial is needed to confirm their hypothesis (Wang et al., 2023a). Vascular endothelial growth factor (VEGF) is a molecule regulating angiogenesis. However, in a tumour context, this is harnessed by the tumour cells to improve tumour angiogenesis and blood vessel accessibility, ultimately supporting tumourigenesis (Shibuya, 2011). A recently completed clinical trial with results published in 2020 showed that the anti-VEGF, bevacizumab, in combination with valproic acid and radiation, was well tolerated but did not improve survival (Su et al., 2020) (NCT00879437). In addition, an ongoing clinical trial will evaluate the survival of DIPG patients when treated with bevacizumab in combination with low-dose radiation (NCT04250064).

Survivin is an inhibitor of the apoptotic pathway and can be found in most cancers (Chen et al., 2016). It is also involved in various cell cycle and signalling pathways such as p53, Wnt, and Notch. Mutations in survivin allow the tumour cells to persist as apoptosis is inhibited (Chen et al., 2016). SurVaxM, a vaccine that immunizes patients with survivin proteins, has been developed. This allows the immune system to recognize survivin-expressing cells as “harmful” and kill the cells, leading to an anti-tumour effect. The clinical trial for SurVaxM is currently in progress and is combined with adjuvant Montanide ISA 51 administration to boost immune responses (NCT04978727). As the hallmark mutation in DIPG, many therapeutic developments have also focused on H3K27M-targetted therapies. This includes the use of H3K27M antigen vaccines (NCT04749641) (Zhang et al., 2023), CAR-T cells (Wang et al., 2023b), and combination therapies with immune checkpoint blockades (NCT02960230) (Grassl et al., 2023). Preliminary data from the NCT04749641 trial testing H3K27M antigen vaccines indicates that survival may be improved compared to other therapies (Zhang et al., 2023). Anti-H3.3K27M CAR-T cells that are specific to HLA-A∗02:01 have recently been developed (Wang et al., 2023b). HLAs, also known as human leukocyte antigens, are proteins that bind to peptides and subsequently present them on the cell surface for T cell recognition (Kulski et al., 2022). In this case, the CAR-T cells would only be able to recognize the H3.3K27M peptide in the context of the HLA-A∗02:01 complex. Wang et al. (2023b) evaluated the binding and recognition capacity of the CAR-T cells using DIPG cell lines. No binding was detected, and upon further investigation, they found that H3.3K27M peptides were not endogenously presented on HLA-A∗02:01 complexes in the DIPG cell lines. The authors concluded that anti-H3.3K27M CAR-T cells specific to HLA-A∗02:01 would not be a feasible immunotherapy for DIPG (Wang et al., 2023b). An ongoing clinical trial is investigating the safety and immune activity of a combination therapy using the H3.3.K27M peptide vaccine, poly-ICLC, and the PD-1 blockade antibody nivolumab. Poly-ICLC acts as an adjuvant to boost immune activity. At the same time, nivolumab can block immunosuppressive signalling, which should enhance the anti-tumour activity induced by the H3.3.K27M peptide vaccine (NCT02960230). Separately, Grassl et al. (2023) evaluated the combination therapy of H3K27M peptide vaccines with anti-PD-1 in DMG; some participants had tumours in the pons, indicating DIPG. Due to regulations, the authors could not conduct their study using a consistent anti-PD-1 treatment. Thus, the drugs used were based on availability. Overall, results indicate that the combination therapy is both safe and immunogenic. Additionally, peripheral immune activity decreased over time, along with the observed tumour regression, and tumour progression coincided with decreased immune responses (Grassl et al., 2023).

### Other cell-based therapies

Autologous cellular vaccines and cell transfers allow for targeting of tumour- and patient-specific antigens. In cellular vaccines, the immune cells and tumour lysate containing the antigens are derived from patients. The cells are then incubated or “pulsed” with the lysate to improve tumour antigen-specificity and re-infused into the patient (Alaniz et al., 2014; Yan et al., 2020). Currently, the immunogenicity and safety of cellular vaccines have been shown in non-DIPG gliomas (Yan et al., 2020). An ongoing clinical trial (NCT04837547) is focusing on vaccination with autologous dendritic cells that have been pulsed with total tumour messenger ribonucleic acid (TTRNA-DC) derived from the patient, this allows the dendritic cells to become loaded with the antigens and can present them to T and B cells when re-infused into the patient (Yan et al., 2020). The same trial will stimulate autologous T lymphocytes *ex vivo* using total tumour messenger ribonucleic acid (TTRNA-xALT) and similarly transfer back into the patient. This clinical trial will primarily assess the safety and feasibility of these therapies. Separately, another clinical trial also focuses on vaccination with TTRNA-DC, but in combination with the cytokine GM-CSF as an adjuvant to boost immune response (NCT03396575). Benitez-Ribas et al. (2018) also published preliminary results demonstrating the safety of autologous dendritic cells pulsed with tumour lysates derived from allogenic DIPG cell lines (Benitez-Ribas et al., 2018).

There has also been interest in natural killer (NK) cells for cell transfers other than CAR-T cells. NK cells have increased anti-tumour activity compared to T cells in glioblastoma due to T cell targeting mutations in the tumour (Lieberman et al., 2019; Galat et al., 2023). Galat et al., 2023 differentiated human pluripotent stem cells into NK cells and assessed their cytotoxicity *in vitro* using DIPG cell lines. They found that the cells could successfully engraft in peripheral blood samples and are cytotoxic against DIPG cells. A separate study is focusing on AloCELYVIR, a novel therapy where oncolytic Adenovirus-infected bone marrow-derived allogeneic mesenchymal stem cells are transfused into the patient. These cells can enhance anti-tumour responses and transport oncolytic molecules to various tumour sites, enabling the killing of cancer cells in locations that are more difficult to reach with conventional therapy. Assessments for the safety and efficacy of this therapy in DIPG patients are currently in progress (NCT04758533).

### Combination therapies

There are also studies focusing on combination therapies, with the majority involving immune checkpoint blockades and cancer vaccines. A clinical trial evaluated the efficacy of nivolumab (anti-PD-1) with ipilimumab (anti-CTLA-4). However, no significant improvements in survival compared were noted (NCT03130959) (Dunkel et al., 2023). A novel neo-antigen heat shock protein vaccine (rHSC-DIPGVax) targeting different DIPG/DMG neo-epitopes has also been developed. Investigation into the vaccine’s safety and tolerability in combination with balstilimab (anti-PD-1) and zalifrelimab (anti-CTLA-4) is currently ongoing (NCT04943848). Dual checkpoint inhibitors have been shown to improve objective response rates in cervical cancer compared to standard treatments, although only in patients with PD-1-expressing tumours (O'Malley et al., 2022). Several clinical trials have also investigated combination therapies such as bevacizumab (anti-VEGF) with cetuximab (anti-EGF) (NCT01884740), as well as nivolumab (anti-PD-1) with bempegaldesleukin, which is an immunostimulatory IL2 pathway agonist (NCT04730349), but have since been terminated due to low accrual (NCT01884740) and change in business objectives (NCT04730349). Nivolumab with bempegaldesleukin showed efficacy in various solid tumours regardless of the PD-1 expression levels (Diab et al., 2020); this may be a potential area to explore as DIPG typically has low PD-1 presence (Lin et al., 2018).

## Challenges

A major challenge with DIPG treatments is the delivery method, as the BBB restricts the penetrance of drugs, antibodies, or exogenously administered molecules. More than 95 clinical trials attempted to date have shown no improvements in survival for DIPG patients, likely due to insufficient drug delivery to the target site and, as such, unable to reach therapeutic concentrations. Although recent advances may alleviate this issue, novel delivery methods include convection-enhanced delivery (CED), intra-arterial therapy, and magnetic resonance-guided focused ultrasound (MRgFUS) (Haumann et al., 2020; Pandit et al., 2020). CED utilizes hydraulic pressure to deliver drugs through a microcatheter driven by a pump; pressure allows for homogenous drug distribution throughout the tumour. Several clinical trials have verified the safety and feasibility of this method in DIPG. Still, the invasive nature of the microcatheter insertion and drug leakage into non-target areas may be causes for concern, especially for off-target effects in healthy tissue (Zhou et al., 2017). In intra-arterial therapy, the drug is injected into an artery close to the tumour, followed by a hyperosmolar drug to open the BBB (Haumann et al., 2020; Pandit et al., 2020). An issue with this method is that the opening of the BBB is non-selective and dependent on the brain region, which allows for the passage of other agents as well as increases in brain fluid due to hyperosmotic-like conditions and impaired homeostasis of endothelial cells (Ikeda et al., 2002; Pandit et al., 2020). These side effects may lead to neurological deficits and toxicity (Pandit et al., 2020). However, conflicting evidence indicates that intra-arterial therapy is safe and feasible (Uluc et al., 2022). MRgFUS is a non-invasive method in which microbubbles are intravenously injected and oscillate upon encountering a focused ultrasound field; allowing for safe and transient opening of the BBB (Alli et al., 2018; Haumann et al., 2020; Pandit et al., 2020). The ultrasound beam can be targeted specifically to cover the tumour location, thus minimizing off-target effects. Various therapeutics can be delivered using MRgFUS, including chemotherapy agents, nanoparticles, antibodies, and gene vectors (Haumann et al., 2020). Safety, feasibility, and efficacy using chemotherapy agents have been demonstrated in DIPG mouse models (Alli et al., 2018; Englander et al., 2021; Ishida et al., 2021). In addition, our group has recently initiated a clinical trial evaluating the delivery of doxorubicin, a BBB-excluded drug, to DIPG patients using MRgFUS (NCT05615623). Regarding immunotherapy, MRgFUS has also been shown to affect and modulate the immune system (Kovacs et al., 2017; Choi et al., 2022), although further investigation is needed to characterize the immune changes in DIPG.

## Conclusion

The numerous studies and clinical trials focusing on immunotherapeutics in DIPG open up exciting possibilities for the future. Drug delivery methods can also be assessed in combination with immunotherapeutic approaches to maximize safety and efficacy in prolonging the survival of DIPG patients.

## References

[B1] Alaniz L. Rizzo M. M. Mazzolini G. (2014). Pulsing dendritic cells with whole tumor cell lysates. Methods Mol. Biol. 1139, 27–31. 10.1007/978-1-4939-0345-0_3 24619667

[B2] Alli S. Figueiredo C. A. Golbourn B. Sabha N. Wu M. Y. Bondoc A. (2018). Brainstem blood brain barrier disruption using focused ultrasound: a demonstration of feasibility and enhanced doxorubicin delivery. J. Control Release 281, 29–41. 10.1016/j.jconrel.2018.05.005 29753957 PMC6026028

[B3] Ausejo-Mauleon I. Labiano S. De La Nava D. Laspidea V. Zalacain M. Marrodan L. (2023). TIM-3 blockade in diffuse intrinsic pontine glioma models promotes tumor regression and antitumor immune memory. Cancer Cell 41, 1911–1926.e8. 10.1016/j.ccell.2023.09.001 37802053 PMC10644900

[B4] Benitez-Ribas D. Cabezon R. Florez-Grau G. Molero M. C. Puerta P. Guillen A. (2018). Immune response generated with the administration of autologous dendritic cells pulsed with an allogenic tumoral cell-lines lysate in patients with newly diagnosed diffuse intrinsic pontine glioma. Front. Oncol. 8, 127. 10.3389/fonc.2018.00127 29755954 PMC5932163

[B5] Cacciotti C. Choi J. Alexandrescu S. Zimmerman M. A. Cooney T. M. Chordas C. (2020). Immune checkpoint inhibition for pediatric patients with recurrent/refractory CNS tumors: a single institution experience. J. Neurooncol 149, 113–122. 10.1007/s11060-020-03578-6 32627129

[B6] Chen X. Duan N. Zhang C. Zhang W. (2016). Survivin and tumorigenesis: molecular mechanisms and therapeutic strategies. J. Cancer 7, 314–323. 10.7150/jca.13332 26918045 PMC4747886

[B7] Chen Y. Zhao C. Li S. Wang J. Zhang H. (2023). Immune microenvironment and immunotherapies for diffuse intrinsic pontine glioma. Cancers (Basel) 15, 602. 10.3390/cancers15030602 36765560 PMC9913210

[B8] Choi H. J. Han M. Seo H. Park C. Y. Lee E. H. Park J. (2022). The new insight into the inflammatory response following focused ultrasound-mediated blood-brain barrier disruption. Fluids Barriers CNS 19, 103. 10.1186/s12987-022-00402-3 36564820 PMC9783406

[B9] Diab A. Tannir N. M. Bentebibel S. E. Hwu P. Papadimitrakopoulou V. Haymaker C. (2020). Bempegaldesleukin (NKTR-214) plus nivolumab in patients with advanced solid tumors: phase I dose-escalation study of safety, efficacy, and immune activation (PIVOT-02). Cancer Discov. 10, 1158–1173. 10.1158/2159-8290.CD-19-1510 32439653

[B10] Dunkel I. J. Doz F. Foreman N. K. Hargrave D. Lassaletta A. Andre N. (2023). Nivolumab with or without ipilimumab in pediatric patients with high-grade CNS malignancies: safety, efficacy, biomarker, and pharmacokinetics-CheckMate 908. Neuro Oncol. 25, 1530–1545. 10.1093/neuonc/noad031 36808285 PMC10398811

[B11] Englander Z. K. Wei H. J. Pouliopoulos A. N. Bendau E. Upadhyayula P. Jan C. I. (2021). Focused ultrasound mediated blood-brain barrier opening is safe and feasible in a murine pontine glioma model. Sci. Rep. 11, 6521. 10.1038/s41598-021-85180-y 33753753 PMC7985134

[B12] Fleischhack G. Massimino M. Warmuth-Metz M. Khuhlaeva E. Janssen G. Graf N. (2019). Nimotuzumab and radiotherapy for treatment of newly diagnosed diffuse intrinsic pontine glioma (DIPG): a phase III clinical study. J. Neurooncol 143, 107–113. 10.1007/s11060-019-03140-z 30830679

[B13] Fujisaki H. Hashii Y. Terashima K. Goto H. Horibe K. Sugiyama K. (2018). PDCT-09. Phase 1/2 Study of DSP-7888 in Pediatric Patients with Malignant Glioma. Neuro-Oncology 20, vi202. 10.1093/neuonc/noy148.839

[B14] Galat Y. Du Y. Perepitchka M. Li X.-N. Balyasnikova I. V. Tse W. T. (2023). *In vitro* vascular differentiation system efficiently produces natural killer cells for cancer immunotherapies. OncoImmunology 12, 2240670. 10.1080/2162402X.2023.2240670 37720687 PMC10501168

[B15] Galon J. Bruni D. (2019). Approaches to treat immune hot, altered and cold tumours with combination immunotherapies. Nat. Rev. Drug Discov. 18, 197–218. 10.1038/s41573-018-0007-y 30610226

[B16] Grassl N. Poschke I. Lindner K. Bunse L. Mildenberger I. Boschert T. (2023). A H3K27M-targeted vaccine in adults with diffuse midline glioma. Nat. Med. 29, 2586–2592. 10.1038/s41591-023-02555-6 37735561 PMC10579055

[B17] Haumann R. Videira J. C. Kaspers G. J. L. Van Vuurden D. G. Hulleman E. (2020). Overview of current drug delivery methods across the blood-brain barrier for the treatment of primary brain tumors. CNS Drugs 34, 1121–1131. 10.1007/s40263-020-00766-w 32965590 PMC7658069

[B18] He X. Xu C. (2020). Immune checkpoint signaling and cancer immunotherapy. Cell Res. 30, 660–669. 10.1038/s41422-020-0343-4 32467592 PMC7395714

[B19] Hwang E. Onar A. Young-Poussaint T. Mitchell D. Kilburn L. Margol A. (2018). IMMU-09. Outcome of patients with recurrent diffuse intrinsic pontine glioma (DIPG) treated with pembrolizumab (anti-PD-1): a pediatric brain tumor consortium study (PBTC045). Neuro-Oncology 20, i100. 10.1093/neuonc/noy059.325

[B20] Ikeda M. Bhattacharjee A. K. Kondoh T. Nagashima T. Tamaki N. (2002). Synergistic effect of cold mannitol and Na(+)/Ca(2+) exchange blocker on blood-brain barrier opening. Biochem. Biophys. Res. Commun. 291, 669–674. 10.1006/bbrc.2002.6495 11855842

[B21] INTERNATIONAL AGENCY FOR RESEARCH ON CANCER (2022) WHO classification of tumours of the central nervous system. Lyon, France: IARC.

[B22] Ishida J. Alli S. Bondoc A. Golbourn B. Sabha N. Mikloska K. (2021). MRI-guided focused ultrasound enhances drug delivery in experimental diffuse intrinsic pontine glioma. J. Control Release 330, 1034–1045. 10.1016/j.jconrel.2020.11.010 33188825

[B23] Johnson T. S. Macdonald T. J. Pacholczyk R. Aguilera D. Al-Basheer A. Bajaj M. (2023). Indoximod-based chemo-immunotherapy for pediatric brain tumors: a first-in-children phase I trial. Neuro Oncol. 26, 348–361. 10.1093/neuonc/noad174 PMC10836763 37715730

[B24] Johung T. B. Monje M. (2017). Diffuse intrinsic pontine glioma: new pathophysiological insights and emerging therapeutic targets. Curr. Neuropharmacol. 15, 88–97. 10.2174/1570159x14666160509123229 27157264 PMC5327455

[B25] Kovacs Z. I. Kim S. Jikaria N. Qureshi F. Milo B. Lewis B. K. (2017). Disrupting the blood-brain barrier by focused ultrasound induces sterile inflammation. Proc. Natl. Acad. Sci. U. S. A. 114, E75–E84. 10.1073/pnas.1614777114 27994152 PMC5224365

[B26] Kulski J. K. Suzuki S. Shiina T. (2022). Human leukocyte antigen super-locus: nexus of genomic supergenes, SNPs, indels, transcripts, and haplotypes. Hum. Genome Var. 9, 49. 10.1038/s41439-022-00226-5 36543786 PMC9772353

[B27] Lieberman N. A. P. Degolier K. Kovar H. M. Davis A. Hoglund V. Stevens J. (2019). Characterization of the immune microenvironment of diffuse intrinsic pontine glioma: implications for development of immunotherapy. Neuro Oncol. 21, 83–94. 10.1093/neuonc/noy145 30169876 PMC6303470

[B28] Li G. Mitra S. S. Monje M. Henrich K. N. Bangs C. D. Nitta R. T. (2012). Expression of epidermal growth factor variant III (EGFRvIII) in pediatric diffuse intrinsic pontine gliomas. J. Neurooncol 108, 395–402. 10.1007/s11060-012-0842-3 22382786 PMC3368992

[B29] Lin G. L. Nagaraja S. Filbin M. G. Suva M. L. Vogel H. Monje M. (2018). Non-inflammatory tumor microenvironment of diffuse intrinsic pontine glioma. Acta Neuropathol. Commun. 6, 51. 10.1186/s40478-018-0553-x 29954445 PMC6022714

[B30] Lindsay H. Onar-Thomas A. Kocak M. Poussaint T. Y. Dhall G. Broniscer A. (2020). EPCT-02. PBTC-051: First In Pediatrics Phase 1 Study of CD40 Agonistic Monoclonal Antibody APX005M in Pediatric Subjects with Recurrent/Refractory Brain Tumors. Neuro-Oncology 22, iii304. 10.1093/neuonc/noaa222.127

[B31] Majzner R. G. Ramakrishna S. Yeom K. W. Patel S. Chinnasamy H. Schultz L. M. (2022). GD2-CAR T cell therapy for H3K27M-mutated diffuse midline gliomas. Nature 603, 934–941. 10.1038/s41586-022-04489-4 35130560 PMC8967714

[B32] Marin-Acevedo J. A. Dholaria B. Soyano A. E. Knutson K. L. Chumsri S. Lou Y. (2018). Next generation of immune checkpoint therapy in cancer: new developments and challenges. J. Hematol. Oncol. 11, 39. 10.1186/s13045-018-0582-8 29544515 PMC5856308

[B33] Mount C. W. Majzner R. G. Sundaresh S. Arnold E. P. Kadapakkam M. Haile S. (2018). Potent antitumor efficacy of anti-GD2 CAR T cells in H3-K27M(+) diffuse midline gliomas. Nat. Med. 24, 572–579. 10.1038/s41591-018-0006-x 29662203 PMC6214371

[B34] Nazha B. Inal C. Owonikoko T. K. (2020). Disialoganglioside GD2 expression in solid tumors and role as a target for cancer therapy. Front. Oncol. 10, 1000. 10.3389/fonc.2020.01000 32733795 PMC7358363

[B35] Oh D. Y. Bang Y. J. (2020). HER2-targeted therapies - a role beyond breast cancer. Nat. Rev. Clin. Oncol. 17, 33–48. 10.1038/s41571-019-0268-3 31548601

[B36] O'Malley D. M. Neffa M. Monk B. J. Melkadze T. Huang M. Kryzhanivska A. (2022). Dual PD-1 and CTLA-4 checkpoint blockade using balstilimab and zalifrelimab combination as second-line treatment for advanced cervical cancer: an open-label phase II study. J. Clin. Oncol. 40, 762–771. 10.1200/JCO.21.02067 34932394 PMC8887945

[B37] Pandit R. Chen L. Gotz J. (2020). The blood-brain barrier: physiology and strategies for drug delivery. Adv. Drug Deliv. Rev. 165-166, 1–14. 10.1016/j.addr.2019.11.009 31790711

[B38] Qi X. W. Zhang F. Wu H. Liu J. L. Zong B. G. Xu C. (2015). Wilms' tumor 1 (WT1) expression and prognosis in solid cancer patients: a systematic review and meta-analysis. Sci. Rep. 5, 8924. 10.1038/srep08924 25748047 PMC4352850

[B39] Shibuya M. (2011). Vascular endothelial growth factor (VEGF) and its receptor (vegfr) signaling in angiogenesis: a crucial target for anti- and pro-angiogenic therapies. Genes Cancer 2, 1097–1105. 10.1177/1947601911423031 22866201 PMC3411125

[B40] Souweidane M. M. Kramer K. Pandit-Taskar N. Zhou Z. Haque S. Zanzonico P. (2018). Convection-enhanced delivery for diffuse intrinsic pontine glioma: a single-centre, dose-escalation, phase 1 trial. Lancet Oncol. 19, 1040–1050. 10.1016/S1470-2045(18)30322-X 29914796 PMC6692905

[B41] Srikanthan D. Taccone M. S. Van Ommeren R. Ishida J. Krumholtz S. L. Rutka J. T. (2021). Diffuse intrinsic pontine glioma: current insights and future directions. Chin. Neurosurg. J. 7, 6. 10.1186/s41016-020-00218-w 33423692 PMC7798267

[B42] Su J. M. Murray J. C. Mcnall-Knapp R. Y. Bowers D. C. Shah S. Adesina A. M. (2020). A phase 2 study of valproic acid and radiation, followed by maintenance valproic acid and bevacizumab in children with newly diagnosed diffuse intrinsic pontine glioma or high-grade glioma. Pediatr. Blood Cancer 67, e28283. 10.1002/pbc.28283 32285998

[B43] Uluc K. Ambady P. Mcintyre M. K. Tabb J. P. Kersch C. N. Nerison C. S. (2022). Safety of intra-arterial chemotherapy with or without osmotic blood-brain barrier disruption for the treatment of patients with brain tumors. Neurooncol Adv. 4, vdac104. 10.1093/noajnl/vdac104 35892048 PMC9307096

[B44] Vitanza N. A. Wilson A. L. Huang W. Seidel K. Brown C. Gustafson J. A. (2023). Intraventricular B7-H3 CAR T cells for diffuse intrinsic pontine glioma: preliminary first-in-human bioactivity and safety. Cancer Discov. 13, 114–131. 10.1158/2159-8290.CD-22-0750 36259971 PMC9827115

[B45] Waldman A. D. Fritz J. M. Lenardo M. J. (2020). A guide to cancer immunotherapy: from T cell basic science to clinical practice. Nat. Rev. Immunol. 20, 651–668. 10.1038/s41577-020-0306-5 32433532 PMC7238960

[B46] Wang S. S. Davenport A. J. Iliopoulos M. Hughes-Parry H. E. Watson K. A. Arcucci V. (2023a). HER2 chimeric antigen receptor T cell immunotherapy is an effective treatment for diffuse intrinsic pontine glioma. Neurooncol Adv. 5, vdad024. 10.1093/noajnl/vdad024 37152812 PMC10158089

[B47] Wang S. S. Pandey K. Watson K. A. Abbott R. C. Mifsud N. A. Gracey F. M. (2023b). Endogenous H3.3K27M derived peptide restricted to HLA-A *02:01 is insufficient for immune-targeting in diffuse midline glioma. Mol. Ther. Oncolytics 30, 167–180. 10.1016/j.omto.2023.08.005 37674626 PMC10477804

[B48] Yan Y. Zeng S. Gong Z. Xu Z. (2020). Clinical implication of cellular vaccine in glioma: current advances and future prospects. J. Exp. Clin. Cancer Res. 39, 257. 10.1186/s13046-020-01778-6 33228738 PMC7685666

[B49] Zhai L. Ladomersky E. Lenzen A. Nguyen B. Patel R. Lauing K. L. (2018). Ido1 in cancer: a Gemini of immune checkpoints. Cell Mol. Immunol. 15, 447–457. 10.1038/cmi.2017.143 29375124 PMC6068130

[B50] Zhang Y. Ji N. Chen G. Wu H. Wang Y. Li X. O. (2023). H3.3-K27M neoantigen vaccine elicits anti-tumor T cell immunity against diffuse intrinsic pontine glioma: the phase I ENACTING trial. J. Clin. Oncol. 41, 2052. 10.1200/jco.2023.41.16_suppl.2052

[B51] Zhang Y. Zheng J. (2020). Functions of immune checkpoint molecules beyond immune evasion. Adv. Exp. Med. Biol. 1248, 201–226. 10.1007/978-981-15-3266-5_9 32185712

[B52] Zhou Z. Singh R. Souweidane M. M. (2017). Convection-enhanced delivery for diffuse intrinsic pontine glioma treatment. Curr. Neuropharmacol. 15, 116–128. 10.2174/1570159x14666160614093615 27306036 PMC5327456

[B53] Zuo P. Li Y. He C. Wang T. Zheng X. Liu H. (2023). Anti-tumor efficacy of anti-GD2 CAR NK-92 cells in diffuse intrinsic pontine gliomas. Front. Immunol. 14, 1145706. 10.3389/fimmu.2023.1145706 37251413 PMC10213244

